# The Circle Pure Rolling Method for Point Cloud Boundary Extraction

**DOI:** 10.3390/s25010045

**Published:** 2024-12-25

**Authors:** Quanou Yang, Zhihui Li, Zhihui Liu, Xinyu Jiang, Xinglong Gao

**Affiliations:** 1 China Aerodynamics Research and Development Center, Mianyang 621000, China; yangqo@mail.ustc.edu.cn (Q.Y.);; 2 School of Aeronautic Science and Engineering, Beihang University, Beijing 100191, China; 3 Facility Design and Instrumentation Institute, China Aerodynamics Research and Development Center, Mianyang 621000, China

**Keywords:** point cloud, boundary extraction, pure rolling, projection

## Abstract

We introduce a circle rolling method (CRM) for boundary extraction from 2D point clouds. The core idea is to create a circle that performs pure rolling on the perimeter of the point cloud to obtain the boundary. For a 3D point cloud, a plane adsorbs points on both sides to create a 2D point cloud, and the CRM is used to extract the boundary points and map them back into space to obtain 3D boundary points. Continuously moving this plane can obtain a complete boundary, which is called the moving adsorption rolling method (MARM). In this paper, we solve the interference problems in our method caused by unidirectional overlapping points and porous structures and successfully validate the solutions in practical examples. Our point cloud boundary extraction method is faster in 2D and better for surface concavities extracted in 3D compared to existing methods, and it is unaffected by sparse points within the point cloud.

## 1. Introduction

There is a complex coupling process involved in the structural response to strong aerodynamic heating during the uncontrolled re-entry of a spacecraft [1]. Our previous work has conducted some research on the structural thermomechanical coupling problem [2,3,4], in which there is an issue that has not been well resolved. When boundary conditions are applied to the structural finite element mesh surface, the efficiency of boundary capture for complex structural shapes is low. If the finite element mesh boundary can be obtained in an extremely short period of time, it can save the preprocessing time of finite element analysis. The structural finite element mesh is a representation of the connectivity of point clouds. When we obtain the boundary points of the point cloud, they can be mapped to the finite element mesh, thereby obtaining the mesh boundary. Our focus in the domain of point cloud boundary extraction is mainly on CAD models, which is different from point cloud models obtained through scanning.

The objects of point cloud boundary extraction in different application situations are images, the 2D point cloud, the 3D geometric structure point cloud, and the 3D building point cloud. Digital images contain color and depth information, and their boundary extraction is often performed using the Canny edge detection algorithm [5]. The RANSAC algorithm [6] is used to extract the external and internal contours at the same time [7], which is represented in the form of lines at the junction of each module of the building point cloud, such as doors, windows, exterior walls, etc., with the original spatial location and size. It is a common method to extract boundary points based on the normal vector estimation of a point cloud, which can identify the boundary features of a scattered point cloud according to the maximum angle between each point and the line connecting the projection points corresponding to its K-nearest neighbors [8,9], and it has the advantage of being fast when the normal direction of the point cloud is known. The convolutional grid architecture can be used to generate point cloud feature lines, which can locate the boundaries of the semantic parts or geometric primitives commonly used in 3D modeling [10], rather than obtaining surface features. In terms of the mesh method, the boundary contour extraction of the building point cloud can be based on the mesh analysis of Delaunay triangulation [7,11]. The *α*-shape technique, derived from Delaunay triangulation [12], is widely applied in extracting the boundary of 2D and 3D point clouds. It is known for its rapid and efficient characteristics, but it is easily neglected for pinch points.

By associating the pixel position of the edge contour in 2D images with the corresponding position of the point cloud data array, the boundary data of the 3D point cloud can be extracted [13]. However, this method has a more complex registration problem for the omni-directional point cloud boundary contour extraction. The depth dimension of the point cloud data of different patches is converted into a 2D image, and the improved Laplace image edge detection method is used to extract the points of each plane, which is also a method that can be used to extend the edge detection method of the 2D image to a 3D point cloud [14], but this method is vulnerable to noise.

An approximate boundary extraction method of point cloud data [15] generates a grid with a certain density according to the view projection of point cloud data, detects the contour of the grid by marking, and finally describes the actual contour of the building with boundary features. However, this method can only obtain the point cloud boundary extraction from a single perspective. Delaunay triangulation is performed on the point cloud data, and the boundary extraction of the point cloud can also be performed according to the judgment of the maximum point-to-point distance threshold of the subdivision edge [16], which can be used to extract the boundary of architectural complexes under aerial view.

A comprehensive linear feature extraction method is used to process 3D objects [17], where the boundary contour can be obtained by using plane projection, radius neighborhood search, etc. Many previous works identify the boundary based on the idea that the points on one side of the adjacent points are boundary points [18,19], which is used to simplify the point cloud and retain the boundary point as much as possible. The scale-invariant 3D point feature method of the boundary point scale space theory is applied to the boundary point feature [20]. By taking the contour observed parallel to the normal line of the viewpoint as the boundary points, the object is rotated continuously, and the boundary points of each orientation of the 3D point cloud can be obtained [21]. The result of this method is the boundary of the point cloud in this view, and the results of each view are not accurately registered, which is suitable for edge detection in automobile driving, but it is difficult to apply to the complete boundary extraction of the solid point cloud.

The *α*-shape technique [12,22], derived from Delaunay triangulation, can be used to extract feature lines from 2D and 3D point clouds [23,24]. The main principle of the α-shape method involves selecting a point, connecting it to a neighboring point, and generating two circumscribed circles that pass through both points and have a radius of *α*. If these two circumscribed circles do not contain any other points, they can be considered as defining a boundary. The ball-pivoting algorithm (BPA) [25], using *α*-shape properties, can produce surface triangles and boundary points on 3D point clouds. The BPA resembles our method, but it differs fundamentally: the former involves rolling a 3D sphere over the surface, where the sphere is typically anchored by three points, thereby obtaining triangular meshes for surface reconstruction, whereas our method consistently employs the rolling of a projected planar circle. The BPA is fast, requires a small amount of memory, and has high surface quality but performs poorly at the specified angle position and is easily affected by internal sparse point sets.

To overcome the limitations mentioned, we use a pure rolling approach to extract the boundaries of 2D and 3D point clouds, which is different from the rolling of the α-shape technique. This method is comparable to touching the surface of an object with a hand, and the place where the finger can reach is the outer boundary of the object. The principle of the circle rolling method (CRM) is that a circle performs clockwise pure rolling on a 2D point cloud. Similar to the “connect-and-slice” idea in reference [26], it is possible to obtain 3D point cloud boundaries using the CRM. Combined with the plane for projection, the moving adsorption rolling method (MARM) can be employed for 3D point cloud boundary extraction.

The following is a summary of our contributions:

(1) We propose the CRM that can quickly extract boundary points from a 2D point cloud without the need to identify each point, and no errors are extracted in the interior away from the point cloud boundary.
(2) We developed the CRM into the MARM using moving adsorption, achieving boundary extraction of the 3D point cloud without significantly increasing time costs due to the increase in the number of points. Similarly, no errors are extracted in the interior away from the point cloud boundary.

## 2. Overview

Our method processes a point cloud, whether it is a 2D or 3D dataset, and then outputs a continuous contour boundary point cloud. For 2D point cloud inputs, we compute its density, determine the size of the rolling circle, find the first rolling point, and perform pure rolling. The return of the rolling circle to the first point signifies the acquisition of the boundary points for that region. For 3D point cloud inputs, we establish a normal plane (also known as an adsorption plane), starting from the minimum value in one direction. A bounding box is constructed around the adsorption plane to slice the point cloud. The points within this area are projected onto the adsorption plane to obtain a 2D point cloud, preserving the mapping relationships. At this point, 2D boundary extraction is performed, mapping the points back into the original 3D space, resulting in the acquisition of the 3D boundary for the sliced area in the current direction. The projection plane is shifted to the next area to obtain a new bounding box, followed by boundary extraction. This process is repeated until boundary extraction is complete for all slices in that direction, culminating in the acquisition of the 3D boundary for that direction. To ensure the boundary is closed, it is necessary to perform the same operations in the other two directions. The results are then merged, and redundant points are removed, resulting in the acquisition of a complete 3D boundary.

**Figure 1:**
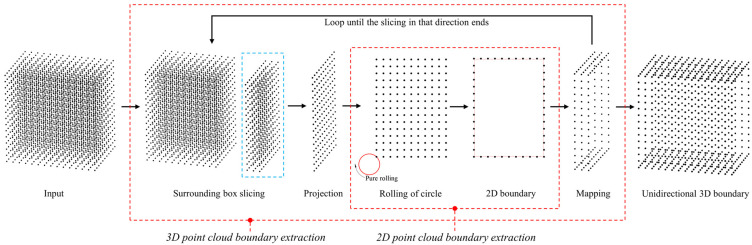
Overview. Our method starts with a 3D point cloud, which includes the independent input part of the 2D point cloud.

An overview of our method is shown in Figure 1. Although the first step in the figure is from a 3D point cloud input, the core of it lies in 2D boundary extraction. Our article starts with 2D point cloud boundary extraction in Section 3.1. The first step is to solve the density of the point cloud (the average value of the distance between each point and its nearest neighbor). The density will be frequently used in the subsequent operations, which will be found in Section 3.1.1. Section 3.1.2 finds the first point and starts circular scrolling to obtain the 2D boundary points. Section 3.1.3 complements Section 3.1.2, which allows for the boundary extraction of non-connected regions. Section 3.2 is the 3D point cloud boundary extraction. Section 3.2.1 uses a bounding box to continuously slice the point cloud, project it to form a 2D point cloud for boundary extraction, and then map it back to the 3D space. Section 3.2.2 complements the 3D point cloud boundary extraction, which resolves the overlapping point problem. Since both our 2D and 3D point cloud boundary extraction methods have complete inputs and outputs, the 2D point cloud experimental part will be found in Section 4.1, and the 3D point cloud experimental part in Section 4.2.

## 3. Methods

### 3.1. The Circle Rolling Method

**Figure 2:**
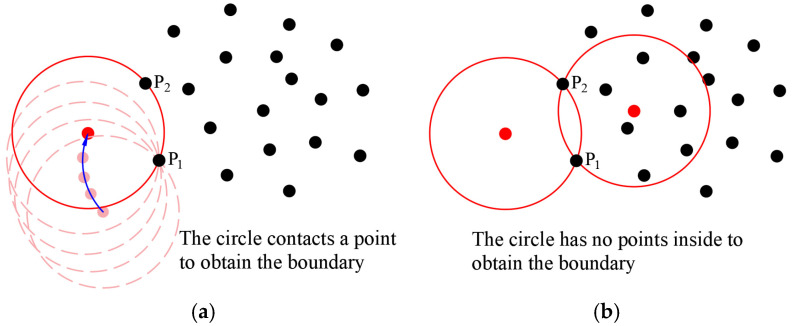
Schematic comparison of boundary point determination between the CMR and *α*-shape: (**a**) CRM; (**b**) *α*-shape.

As shown in Figure 2a, the CRM involves taking a circle and rotating it around a boundary point P_1_; the next boundary point is obtained when it comes into contact with P_2_. The center of the rotating circle traces a rolling path, as indicated by the blue arrows in Figure 2a. In Figure 2b, the *α*-shape method uses points P_1_ and P_2_, along with the radius, to define two circles and checks for the presence of other points within these circles. If one of the circles contains no other points, both points are deemed to be on the boundary. For traversing the objects, the CRM only needs to roll through the boundary points in sequence, whereas the *α*-shape method requires checking all points individually.

In order to solve the boundary of the 2D point cloud using the CRM, it is necessary to obtain the rolling parameters, which include the rolling circle’s radius *r*_o_ and the rolling radius *r*_p_. These two radii depend on the point cloud density *ρ*, which needs to be solved first, and then the boundary points can be obtained through pure rolling step by step.

#### 3.1.1. Calculation of Point Cloud Density

The density of a point cloud is defined as the average distance between each point and its nearest point. It is necessary to traverse each point to find the nearest distance. If all the points are searched, the process becomes cumbersome. To overcome this issue, point cloud data can be organized into a tree data structure, such as kd-Tree [27,28], which allows for fast neighbor lookup. Our method employs a kd-Tree neighbor search of the open source Point Cloud Library (PCL) [29].

Finding the nearest neighbor distance for each point is typically necessary. However, for a large number of points, the computation time may become unacceptable. For our purposes, an accurate density value is not required, and even incomplete sampling can yield satisfactory results, assuming no outliers. The neighbor distance *d_i_* can be obtained by distributed sampling, and then the *ρ* can be calculated, as shown in Formula (1):

(1) $$\left\{\begin{array}{l} n_{s}=\frac{m}{n}\\ \rho =\sum_{i=1}^{n}d_{i}/n \end{array}\right.$$

where *m* is the total number of points in the point cloud, *n* is the number of sampling points, and *n_s_* is the sampling interval; that is, one point is taken at an interval of *n_s_* points to calculate the neighbors, and an appropriate *n* is selected according to the total number of points in the point cloud.

#### 3.1.2. Rolling Steps

**Figure 3:**
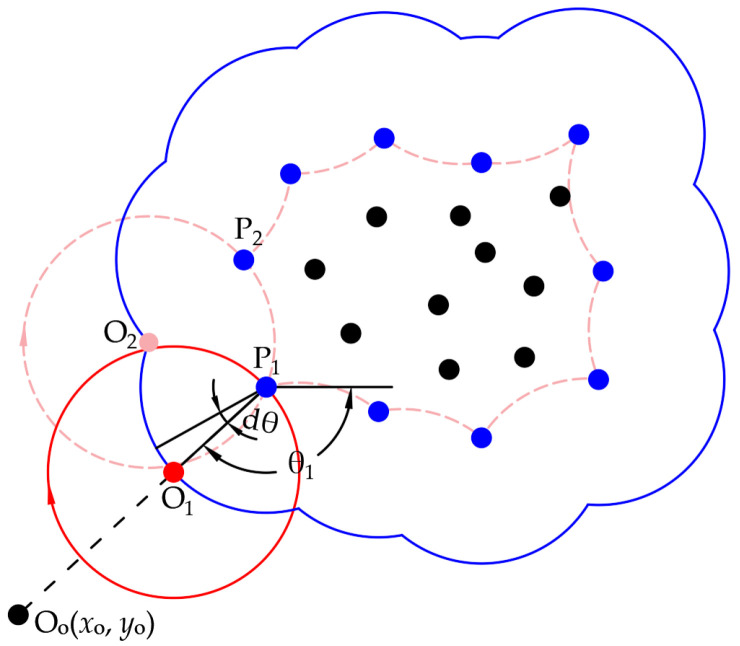
Schematic diagram of the CRM. The red line marks the rolling circle, the light red line shows the paths at boundary contacts, and the blue line shows the path of the circle’s center. Blue points mark the boundary points.

The schematic diagram of the CRM is shown in Figure 3, and the steps are as follows:

(1) Determine the lower-left corner point O_o_ and the starting point P_1_ for rolling. O_o_ will be used to create the rolling starting point, and the coordinates (*x*_o_, *y*_o_) can be obtained from Formula (2). As for P_1_, it is the nearest point to O_o_ in the point cloud and serves as the first boundary point.

(2) $$\left\{\begin{array}{c} x_{o}=x_{\min}-(x_{\max}-x_{\min})\\ y_{o}=y_{\min}-(y_{\max}-y_{\min}) \end{array}\right.$$

where *x*_max_, *x*_min_, *y*_max_, and *y*_min_ represent the maximum and minimum values of each coordinate component of the point cloud.

(2) Determine the values of *r*_o_ and *r*_p_, which denote the radii of the red and blue circles in Figure 3, respectively. The radius *r*_p_ represents the distance at which the center O_1_ of the rolling circle revolves around P_1_. As usual, 0.75*ρ* ≤ *r*_o_ ≤ 4*ρ*, *r*_p_ = *r*_o_. It should be noted that the larger the value of *r*_o_, the lower the curvature of the fitting curve of the boundary point. To prevent the rolling circle from sinking into the interior of the point cloud, which would cause boundary extraction to fail, it is necessary to ensure that *r*_o_ is not less than the point cloud density at that location. However, if *r*_o_ is too large, boundary loss may occur at concave positions. Therefore, by default, *r*_o_ = *ρ*, and it is adjusted based on the occurrence of incorrect point extraction or the loss of boundary points: we increase *r*_o_ if there is incorrect point extraction and decrease *r*_o_ if there is loss of boundary points.

(3) Real-time determination of the rolling circle’s center. The included angle between the line segment P_1_O_1_ and the positive *x*-axis is *θ*_1_; the initial center of circle O_1_ rotates clockwise around P_1_. When the rotation angle of d*θ* is reached, the coordinate of O_1_’s center is transformed. Its calculation is expressed by Formula (3):

(3) $$\left\{\begin{array}{l} \theta_{i+1}=\theta_{i}-d\theta \\ x_{oi+1}=x_{Pj}+r_{o}\cos\theta_{i+1}\\ y_{oi+1}=y_{Pj}+r_{o}\sin\theta_{i+1} \end{array}\right.$$

where (*x*_o*i*_, *y*_o*i*_) are the coordinates of O*_i_*, (*x*_P*j*_, *y*_P*j*_) are the coordinates of P*_j_*. *i* = 1, 2, 3,…until the circle touches the next boundary point, and *j* is the serial number of the boundary point.

(4) Determine whether the boundary of the rolling circle touches the next point or not. Search the nearest neighbor point P_m_(*x*_m*i*_, *y*_m*i*_) of the point O_1_ in the point cloud at each rolling step and judge whether the distance *d* between O_1_ and P_m_ is *r*_o_; that is, if Formula (4) is satisfied, a new boundary point is found.

(4) $$\left\{\begin{array}{l} d=\sqrt{{\left(x_{oi}-x_{mi}\right)}^{2}+{\left(y_{oi}-y_{mi}\right)}^{2}}\\ d\in r_{o}\cdot (1\pm \Delta ) \end{array}\right.$$

where Δ is the tolerance, usually Δ = d*θ*/2. Due to the rotation angle being d*θ*, it is difficult to ensure that *d* = *r*_o_. When *r*_o_ (1 − d*θ*/2) ≤ *d* ≤ *r*_o_ (1 + d*θ*/2), it can compensate for the d*θ* gap during rotation.

(5) Determine whether the rolling circle rolls back to the starting point. If affirmative, the rolling process ends; if negative, revert back to step (3) and continue with the rolling calculation.

**Figure 4:**
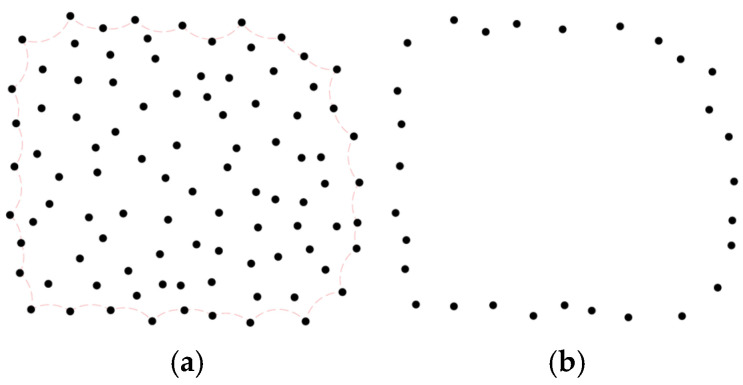
Example of obtaining the point cloud boundary by CRM: (**a**) input point cloud, the light red line indicate the rolling path.; (**b**) boundary extraction result.

Based on the aforementioned five steps, the boundary points for the entire plane’s point cloud can be acquired, as illustrated in Figure 4.

#### 3.1.3. Non-Connected Point Cloud Processing

**Figure 5:**
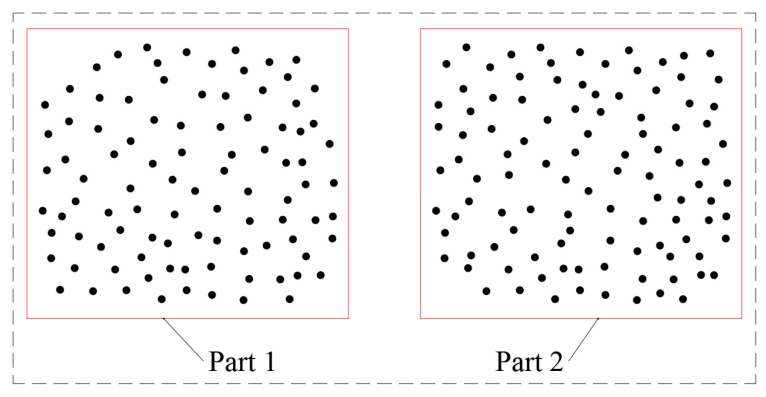
A point cloud contains multiple parts.

A point cloud can have several unconnected regions, as shown in Figure 5. At this time, the CRM prioritizes the boundary extraction of Part 1. Once the boundary closed-loop is formed, the program determines that the boundary extraction of the section has been completed, while Part 2 will not undergo boundary extraction again. Given this shortcoming, it is necessary to further improve the CRM.

In Figure 5, after extracting the boundary of Part 1, it is necessary to determine whether there are other parts to be extracted by the boundary. Therefore, the following implicit boundary judgment method is proposed.

**Figure 6:**
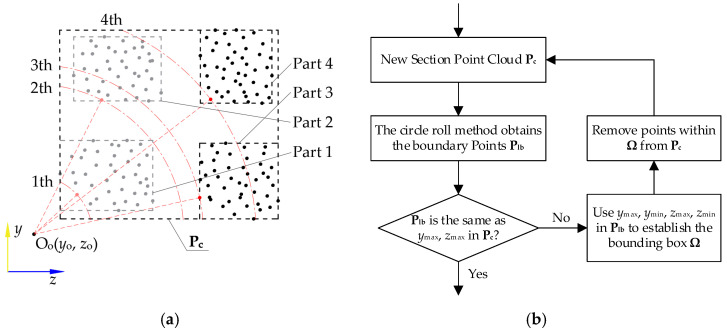
Multi-part point cloud processing of the same section: (**a**) schematic of the processing sequence, where those red lines indicate the closest search ranges for each part from point O_o_; (**b**) flowsheet of treatment method.

As shown in Figure 6, after extracting the boundary points P_lb_ of Part 3, the maximum *y* and *z* values are found. If the values match those of the boundary of the input point cloud P_c_ at this time, it means that the boundary extraction of the section is completed. Otherwise, the maximum *y* and *z* values and the minimum *y* and *z* values in P_lb_ are used to establish a bounding box Ω, and the points in Ω are deleted from P_c_ (as the points in Parts 1 to 2 above have been extracted and discarded, expressed in gray). Among the remaining points, we find the point closest to O_o_. The boundary of Part 3 is extracted again according to the CRM in Figure 3, and we continue with our determinations after the identification is completed until there is no part of the section point cloud remaining.

### 3.2. The Moving Adsorption Rolling Method

The purpose of the moving adsorption is to extend the CRM to the 3D point cloud, just like moving a scan in space. “Adsorption” refers to projecting the points onto a constantly moving plane. When combined with the CRM, the MARM is formed, which can complete the boundary extraction of 3D point clouds.

#### 3.2.1. Plane Moving Adsorption

**Figure 7:**
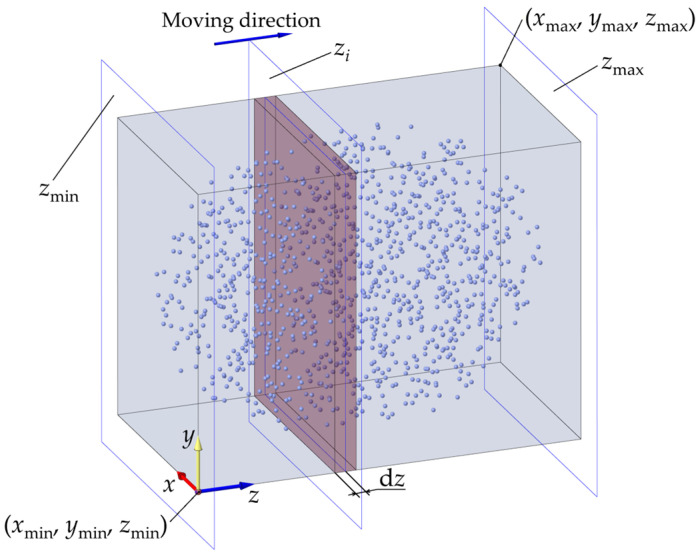
Schematic diagram of moving adsorption.

Plane moving adsorption is the process of slicing a 3D point cloud and then projecting it, as shown in Figure 7. Taking the *z* direction as an example, the *z_i_* (*z*_min_ ≤ *z_i_* ≤ *z*_max_, *i* = 1, 2, 3…) plane is perpendicular to the *z*-axis, and an increase in d*z* by *z_i_* is equivalent to a move, and the points within *z* ± d*z* are projected to the plane; that is, the 2D point cloud P_c_ at the section *z_i_* is obtained. Then, the boundary points are extracted using the CRM, and these boundary points correspond to the original point cloud; that is, the boundary points of the 3D point cloud within *z* ± d*z* are obtained. Until *z_i_* increases from *z*_min_ to *z*_max_, the boundary points of the point cloud in this direction are extracted.

The detailed process of the MARM in the *z* direction is as follows:

(1) Obtain the extreme values *x*_min_, *x*_max_, *y*_min_, *y*_max_, *z*_min_, and *z*_max_ of the three directions of the point cloud and determine the tolerance d*z* of the moving direction, which can be d*z* ∈ [0.5*ρ*, *ρ*]. If the slope of the boundary surface in the *z*-direction is too steep, taking a larger d*z* value will cause the boundary points in space to be inside the plane after projection, leading to the loss of boundaries. Therefore, by default, d*z* is taken as *ρ*, and d*z* is reduced when the slope is steep and increased for faster computation when the slope is gentle.

(2) Obtain *z_i_* according to Formula (5), establish a bounding box with the two diagonal points, (*x*_min_, *y*_min_, *z_i_* − d*z*) and (*x*_max_, *y*_max_, *z_i_* + d*z*), and remove the *z* coordinates of all points within the range to obtain a plane point cloud P_c_ containing only (*x*, *y*), or change all their *z* coordinates to *z_i_*.

*z_i_*_+1_ = *z_i_* + d*z*, (5)

where the subscript *i* denotes the *i*-th movement of the plane, *i* = 1, 2, …, [(*z*_max_ − *z*_min_ )/d*z*].

(3) The CRM is used to extract the boundary of the plane point cloud from step (2), and they are mapped to the original *z* values to obtain the boundary points in the bounding box. If *z_i_* = *z*_max_, the boundary extraction is finished; otherwise, return to step (2).

#### 3.2.2. Overlap Points Processing

**Figure 8:**
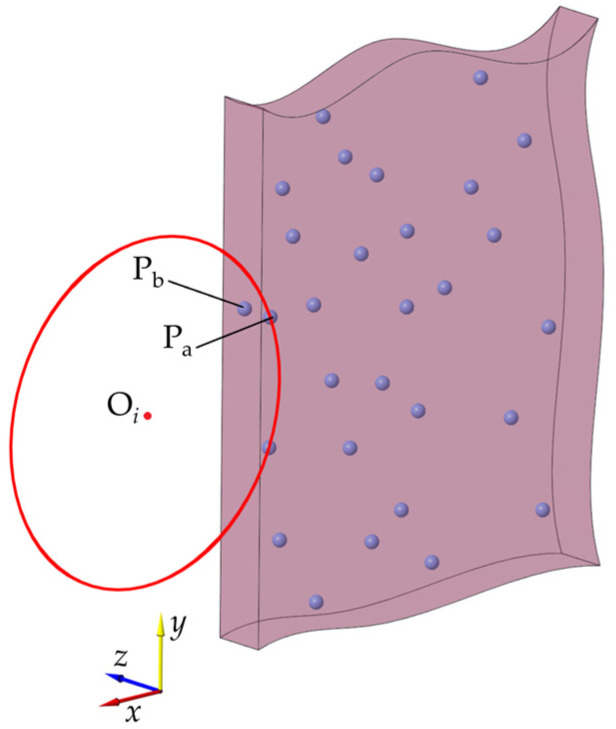
Schematic diagram of overlap points, the red circle represent the rolling circle, and the indigo points represent the point cloud.

The MARM has the ability to extract the boundary points of the general model, but there are some limitations that require improvement. For example, multiple points in a bounding box have the same *x* and *y* but different *z* values; they overlap in the *z* direction. As shown in Figure 8, the points P_a_ and P_b_ are projected to the same point on the *x–y* plane at *z_i_*. When the circle O*_i_* rolls over, it will touch both points at the same time. If P_a_ is recorded as a boundary point, according to the judgment criterion of the CRM in this paper, P_b_ will be identified as the next boundary point when the clockwise roll continues. If this issue is not dealt with, P_a_ will be identified again as a boundary point, leading to the algorithm becoming stuck in an endless loop between P_a_ and P_b_, ultimately causing the failure of boundary extraction.

**Figure 9:**
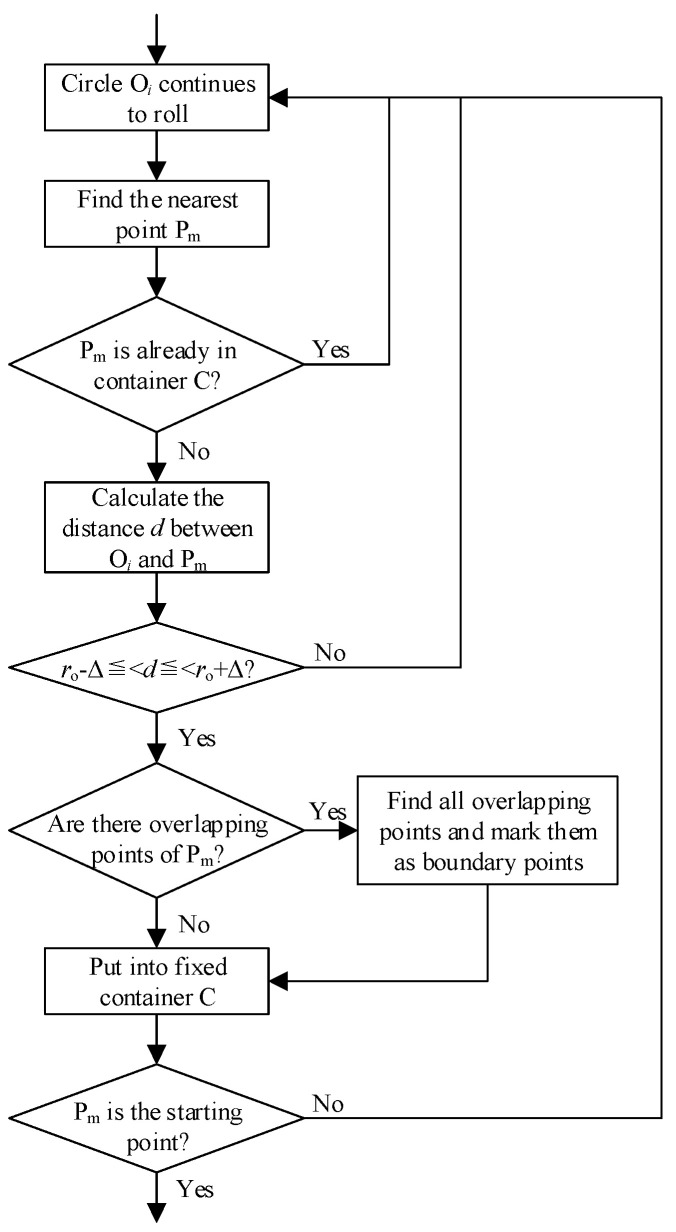
Overlapping point processing.

The problem-solving method involves the following steps: if the nearest neighbor P_m_ is identified as a boundary point, and its *x* and *y* values are the same as those of the second nearest neighbor, we determine whether more points in the bounding box overlap here, record them as boundary points, and then directly enter the next step of the rolling process. To make the rolling process more reliable, it is necessary to establish a container C that tracks the serial numbers of the boundary points that have already been searched. In circle rolling, we check if the serial number of a new boundary point is in C and then proceed directly to the next rolling step without adding new points if it is. The size of C should be limited to ensure that these comparisons do not hinder operation efficiency. Figure 9 illustrates the operation process.

**Figure 10:**
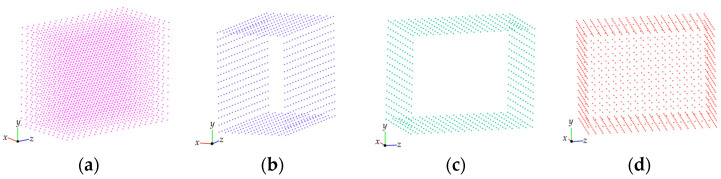
Extracting boundaries from a cuboid point cloud: (**a**) cuboid point cloud; (**b**) result in the *z*-direction; (**c**) result in the *x*-direction; (**d**) merged result.

There is also a general case where the *z* values of many points in the 3D point cloud are equal to *z*_min_ (or *z*_max_), and they are indeed boundaries, such as the cuboid point cloud shown in Figure 10a. In such cases, the MARM only extracts boundary points in the *z* direction, resulting in a large hole, as shown in Figure 10b.

To fill the hole, it is necessary to perform a MARM operation from the *x*-axis (or *y*-axis) to obtain the boundary points in this direction, as shown in Figure 10c. There will also be holes in the two surfaces. At this time, the points in Figure 10b,c are merged, and then the duplicate points are removed. The integrated result is shown in Figure 10d, which realizes the complete boundary extraction of six faces in the special case of a cuboid.

## 4. Results and Discussion

### 4.1. Two-Dimensional Point Cloud Boundary Extraction

**Figure 11:**
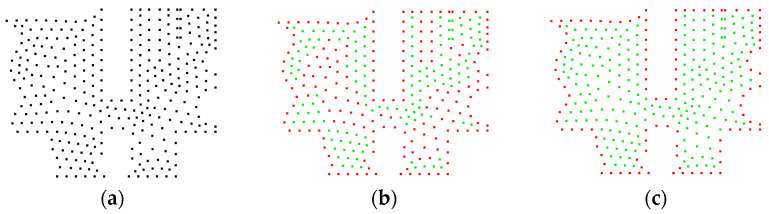

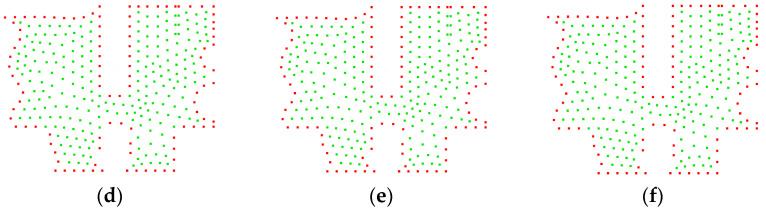
Boundary extraction results for an irregular point cloud: (**a**) the 2D input point cloud; (**b**) the *α*-shape result at *α* = 6*ρ*, with 246 points extracted; (**c**) the *α*-shape result at *α* = 10*ρ*, with 105 points extracted; (**d**) the *α*-shape result at *α* = 15*ρ*, with 101 points extracted; (**e**) the result of the CRM at *r*_o_ = 1.5*ρ*, with 105 points extracted; (**f**) the result of the CRM at *r*_o_ = 2*ρ*, with 101 points extracted.

The CRM is compared with the *α*-shape algorithm from the Computational Geometry Algorithms Library (CGAL) [30] for extracting boundaries from 2D point clouds. An irregularly shaped point cloud, as shown in Figure 11a, is used as the input. The output results are shown in Figure 11b–f. For the sake clarity, the extracted boundary point cloud (red) covers the original point cloud (green). Complete encirclement of the green points by the red points indicates excellent extraction quality. Encirclement of the red points by the green points indicates a problem. The presence of green points on the boundary indicates areas where no boundary is extracted.

Tests have shown that the *α* of the *α*-shape algorithm must be correlated with the point cloud density; otherwise, its determination becomes difficult. In this study, the density from the CRM is used to perform a linear transformation to determine the input parameters for both methodologies. Figure 11b shows the results of the *α*-shape algorithm with *α* = 6*ρ*, revealing instances of erroneous extraction internally. Increasing *α* to 10*ρ*, as depicted in Figure 11c, yielded optimal recognition without any false extractions. Further increasing *α* to 15, as shown in Figure 11d, led to a decrease in the number of boundary points. When employing the CRM for extraction, the best recognition performance is achieved at *r*_o_ = 1.5*ρ*, as shown in Figure 11e, with results fully consistent with Figure 11c. Increasing *r*_o_ to 2*ρ* in the CRM also led to a reduction in boundary points. This demonstrates that for boundary extraction in 2D point clouds, both the CRM and the *α*-shape algorithm require parameter adjustments for superior boundary extraction. Both methods can produce equally excellent boundary extraction quality.

**Figure 12:**
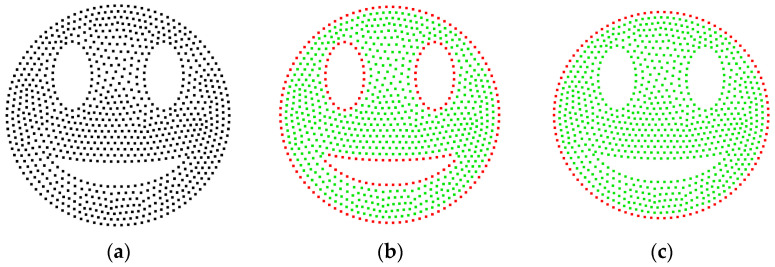
Boundary extraction results for a smiling face point cloud: (**a**) the 2D input point cloud; (**b**) the *α*-shape results at *α* = 4*ρ*, with 195 points extracted; (**c**) the result of the CRM at *r*_o_ = 2*ρ*, also with 102 points extracted.

To validate the effectiveness of inner and outer boundary extraction, the *α*-shape algorithm and the CRM are used to delineate the boundaries of the smiley face point cloud depicted in Figure 12a. Figure 12b shows that the *α*-shape algorithm can identify the eyes and mouth of the smiley face, with the inner boundary being under-extracted at the corners of the mouth, while the outer contour is fully extracted. On the other hand, Figure 12c shows that the CRM only fully extracts the outer boundary, neglecting the inner contour. Although this may seem like a limitation in this particular case, it could potentially be an advantage in other contexts.

**Figure 13:**
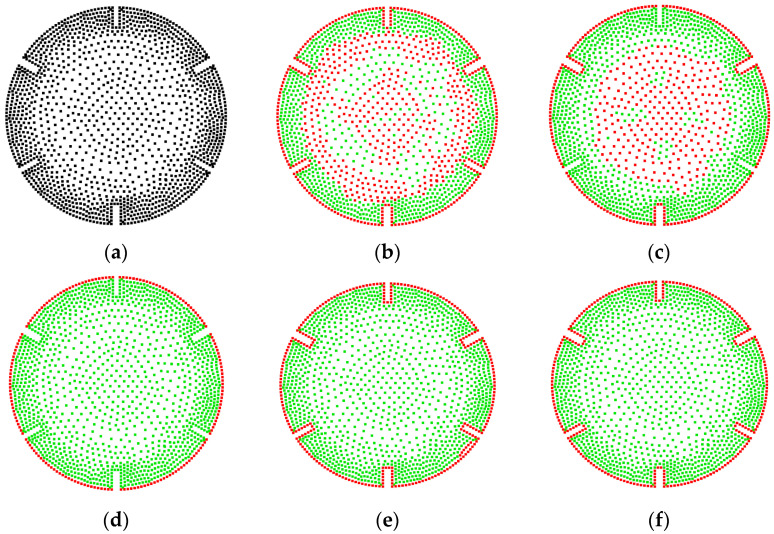
Boundary extraction results for a ratchet point cloud: (**a**) the 2D input point cloud; (**b**) the *α*-shape results at *α* = 2.4*ρ*, with 887 points extracted; (**c**) the *α*-shape results at *α* = 4*ρ*, with 668 points extracted; (**d**) the *α*-shape results at *α* = 7.8*ρ*, with 186 points extracted; (**e**) the results of the CRM at *r*_o_ = 0.5*ρ*, with 273 points extracted; (**f**) the results of the CRM at *r*_o_ = 0.81*ρ*, with 258 points extracted.

The *α*-shape algorithm and the CRM are used to extract the boundary of the ratchet point cloud in Figure 13a, which has a relatively sparse interior. In Figure 13b, with *α* = 2.4*ρ*, the *α*-shape algorithm successfully extracted the complete outer contour but misidentified a significant number of non-boundary points internally. *α* = 4*ρ* can mitigate internal misidentification but at the cost of losing the boundary from three small gaps, as shown in Figure 13c, which still fails to eliminate internal interferences entirely. To prevent misidentifications with the *α*-shape algorithm, the result when *α* is set to 7.8*ρ* is shown in Figure 13d, where none of the six gaps along the boundary are effectively extracted. Figure 13e shows the superior extraction of the external boundary achieved by the CRM with *r*_o_ set to 0.5*ρ*, although local misidentifications occur in the lower right corner of the ratchet. Increasing *r*_o_ to 0.81*ρ* eliminated the misidentifications, and Figure 13f shows the relatively complete boundary achieved by our approach. Clearly, the CRM significantly outperforms the *α*-shape algorithm in the extraction of such models.

**Figure 14:**
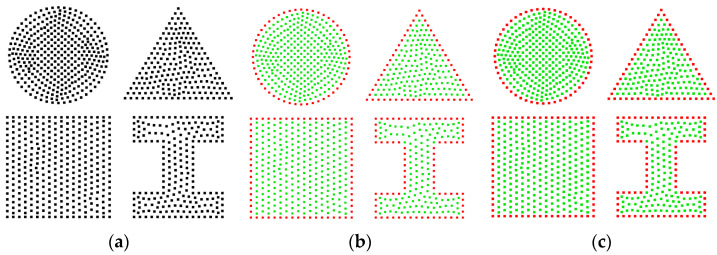
Boundary extraction results for a non-connected point cloud: (**a**) the 2D input point cloud; (**b**) the *α*-shape results at *α* = 5*ρ*, with 254 points extracted; (**c**) the results obtained by the CRM at *r*_o_ = 1.3*ρ*, with the same number of 254 points extracted.

The *α*-shape algorithm and the CRM are used to extract the boundary of the non-connected point cloud shown in Figure 14a, which includes four regions. Figure 14b shows that the α-shape algorithm, with *α* set to 5*ρ*, successfully extracted the outer contours with the most integrity. Similarly, Figure 14c shows the result of the CRM at a threshold of *r*_o_ = 1.3*ρ*, which is in perfect agreement with the results obtained by the *α*-shape algorithm. This supports the effectiveness of the CRM in extracting the boundaries of point clouds with non-connected regions.

We use Formula (6) to calculate the boundary extraction accuracy and error rate. The calculation results for the aforementioned four point cloud models are shown in Table 1, reflecting that the CRM has an extremely low error rate, and its outer boundary extraction rate is not worse than that of the alpha-shape.

(6) $$\left\{\begin{array}{l} k_{bdry}=\frac{n_{cor}}{n_{ref}}\\ k_{error}=\frac{n_{ext}-n_{cor}}{n_{total}} \end{array}\right.$$

where $k_{bdry}$ represents the boundary extraction accuracy, $n_{ext}$ is the number of extracted points, $n_{ref}$ is the number of reference boundary points, $k_{error}$ is the error rate, $n_{cor}$ is the number of correctly extracted boundary points, and $n_{total}$ is the total number of points.

**Figure 15:**
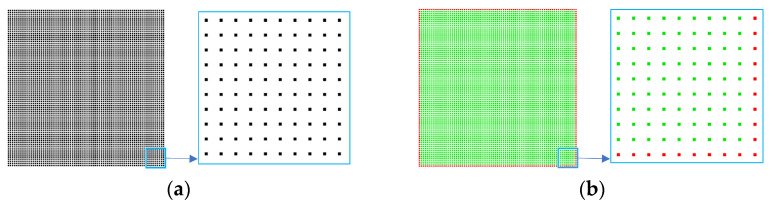
Boundary extraction results for a 6561 (81 × 81) square point cloud: (**a**) the 2D input point cloud; (**b**) the *α*-shape is taken as *α* = 20*ρ* and *α* = 200*ρ*, respectively, and the CRM is taken as *r*_o_ = *ρ* and *r*_o_ = 2*ρ*, respectively, both of which obtain the same result, which is 320 boundary points.

To compare the speed of the CRM with the *α*-shape algorithm, boundary extraction is performed on a uniform point cloud of squares using both methods. The *α*-shape algorithm is set to *α* = 20*ρ* and *α* = 200*ρ*, respectively, while the CRM used *r*_o_ = *ρ* and *r*_o_ = 2*ρ*. As shown in Figure 15a for a point cloud of 6561 (81 × 81) points, both methods produced results as shown in Figure 15b. Boundary extraction is also performed on point clouds of size 40,401 (201 × 201), 160,801 (401 × 401), 641,601 (801 × 801), and 4,004,001 (2001 × 2001). The extraction results for each type of point cloud are fully consistent between the two methods. Due to the excessive density of points, the results are not shown here but are documented in Table 2.

**Figure 16:**
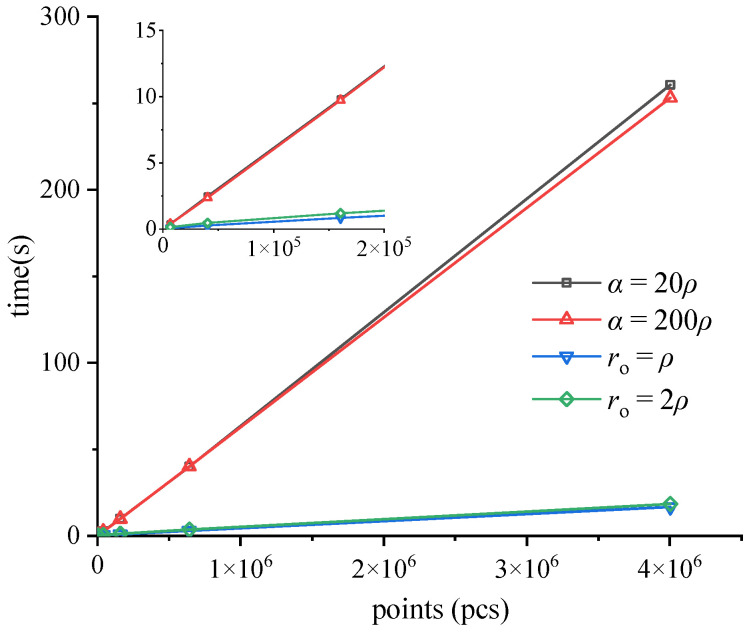
The relationship between input points and time consumption in Table 2. The *α* value in the legend represents the *α*-shape algorithm, while the *r*_o_ value represents the CRM.

Based on the input point numbers and time consumption results presented in Table 2, a line graph is constructed, as shown in Figure 16. The time taken by the *α*-shape is directly proportional to the number of points, with negligible variations observed when selecting different *α* values. The time taken by the CRM is directly proportional to the number of points; the larger the *r*_o_, the longer the time taken, but there is no significant difference. In terms of boundary extraction for identical 2D point clouds, the CRM outperforms the *α*-shape in time efficiency, with a significantly smaller linear coefficient. The superior efficiency of the CRM becomes even more pronounced when dealing with superscale point clouds.

### 4.2. Three-Dimensional Point Cloud Boundary Extraction

#### 4.2.1. Method Testing

In order to verify that the MARM can adapt to more complex models and find the shortcomings of the method, boundary extraction is performed for three typical point cloud models.

**Figure 17:**
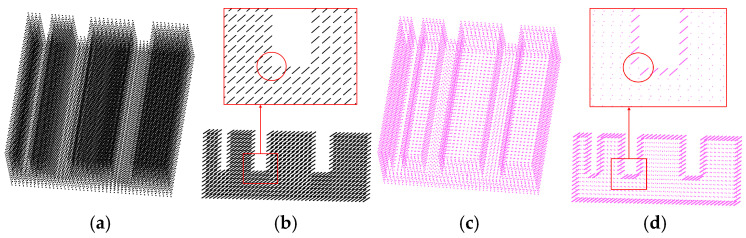
Boundary extraction of a slit model: (**a**) input; (**b**) detailed view of input; (**c**) result; (**d**) detailed view of result. The red circles indicate the highlighted details at the comparison location, with subsequent figures being similar.

**Figure 18:**
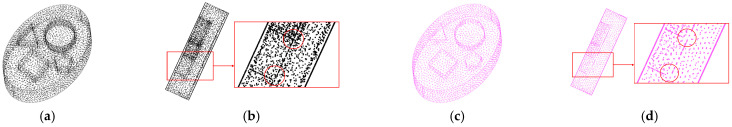
Initial groove model: (**a**) input; (**b**) detailed view of input; (**c**) result; (**d**) detailed view of result.

(1) The first model is a slit model, as shown in Figure 17. Comparing the red circle position in Figure 17b,d, a hole appears in the corner, which is a right angle position where the rolling circle cannot touch the edge line. This can be improved by reducing the radius of the rolling circle.

(2) The second model is a groove model, as shown in Figure 18. At the bottom of the groove in Figure 18d is a projection view; this position should have obvious T-shaped traces, as shown in Figure 18b, but there are missing boundary points, and it is still difficult to make up for the loopholes by rolling from the three directions of the coordinate axis. The bottom of the rest of the grooves also has a similar problem, which is the same reason as the slit model’s defect. In the case of ensuring the overall extraction quality, reducing the rolling circle radius *r*_o_ can improve the quality of the groove bottom boundary extraction.

**Figure 19:**
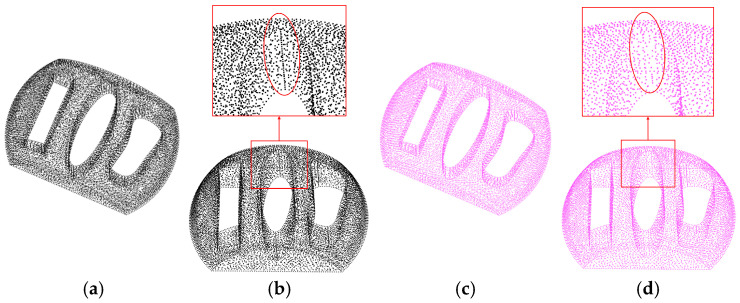
Initial through-hole model: (**a**) input; (**b**) detailed view of input; (**c**) result; (**d**) detailed view of result.

The third model is a through-hole model, as shown in Figure 19. The boundary extraction result of this model is basically complete. There is a column of relatively regular points in the red box of Figure 19b, but the boundary extraction result of Figure 19d lacks this column of points. The main cause of this problem is that the moving distance d*z* is too large. When the *z_i_* plane passes through these points, other points within the range of ±d*z* are located at the periphery of these points on the projection plane. The extraction of boundary points follows the principle of periphery priority. On the premise of global extraction, this result can be improved by appropriately reducing the value of d*z*.

According to the above analysis, the MARM used for 3D point cloud boundary extraction has the following advantages:

(1) It does not involve complex mathematical calculation problems and is easy to realize.
(2) The outer surface of a general shape is almost completely extracted.
(3) It can be applied to shapes with external sharp corners, holes, grooves, etc.

Our method also has drawbacks: it is difficult to handle the internal sharp corners of the model perfectly. The CRM rolls on the surface of the point cloud in a circular manner, which cannot touch the deepest part of the slot like a human hand.

#### 4.2.2. Method Comparison

Common methods for boundary extraction from 3D point clouds include the *α*-shape technique [12,22] and the normal vector estimation method [8,31]. For comparison, we use the PCL’s *α*-shape method, which still requires only the adjustment of *α* to obtain 3D boundaries of varying quality. We use the normal vector estimation method in PCL, which is referred to as the normal method in the following text. It is required to set a normal estimation radius *r*_n_, a boundary estimation radius *r*_b_, and a boundary estimation angle threshold *θ*_t_.

It is worth noting that we use a pre-existing reference boundary point cloud for our results evaluation, which is consistent with the input model surface boundary point set. The evaluation is conducted using a point cloud cover display, resulting in two evaluation images per result. The first image shows the extracted boundary (red) covering the reference boundary (green), with a higher proportion of red indicating a higher number of correctly identified boundary points. The second image shows the reference boundary (green) covering the extracted boundary (red), where a smaller proportion of red signifies a lower error rate. The following results are presented in this image format.

**Figure 20:**
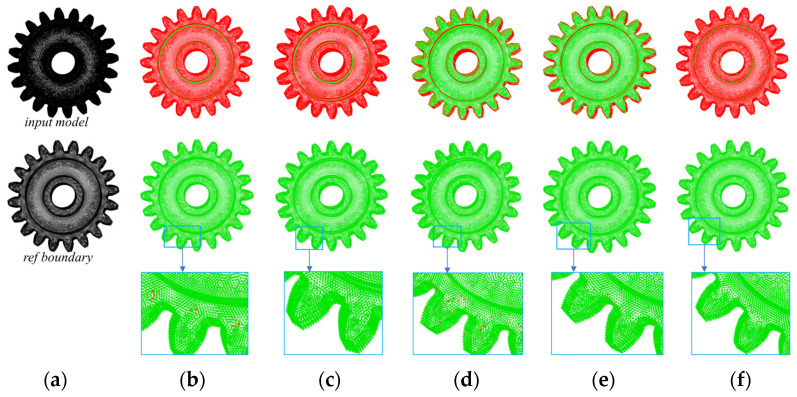
Boundary extraction for the gear point cloud: (**a**) the 3D input point cloud, where the upper image is the input for three methods and the bottom image is the reference boundary of the point cloud; (**b**) the *α*-shape method with *α* = 1.7*ρ*, where the upper image shows the result point cloud (red) masking the reference point cloud (green), and the lower points represent the reference point cloud masking the result point cloud; (**c**) the *α*-shape method with *α* = 2*ρ*; (**d**) the normal method with parameters *r*_b_ =2.5*ρ*, *r*_n_ = 30, and *θ*_t_ = pi/2; (**e**) the normal method with parameters *r*_b_ =3*ρ*, *r*_n_ = 30*ρ*, and *θ*_t_ = pi/2; (**f**) the MARM with *r*_o_ = 2*ρ*.

During the boundary extraction process, it is crucial to minimize the occurrence of false points (non-boundary points), which may sometimes require the sacrifice of a certain number of accurate boundary points. We use the *α*-shape method, the normal method, and the MARM on the 3D gear point cloud shown in Figure 20. The *α*-shape method and the MARM require the adjustment of a single parameter. However, the normal method requires the coordination of three parameters.

We use these three methods to extract boundaries from the gear point cloud shown in Figure 20a. As shown in Figure 20b, when the *α*-shape parameter is set to *α* = 1.7*ρ*, a count of extracted points is achieved; however, there are few sporadic erroneous points, but the extraction result remains satisfactory. Increasing *α* further reduces the number of erroneous points but also reduces the number of points extracted. Figure 20c shows that *α* = 2*ρ* is a reasonable parameter for the *α* method, resulting in fewer extracted points than the previous two cases, but no errors. The normal method requires the adjustment of three parameters: first, an appropriate *r*_n_ is required, which, after several adjustments, is found to be 30 times the density, followed by *θ*_t_ set to pi/2. Figure 20d shows that *r*_b_ = 2.5*ρ* captures more of the tooth surface and shaft contact surface contours but hardly achieves good detection in the axial vertical plane and has a high number of false points. In Figure 20e, *r*_b_ = 3*ρ* significantly reduces the number of false points, but it does not improve the extraction quality. The MARM, using a default radius of *r*_o_ = 1.5*ρ*, achieves the results shown in Figure 20f, where most of the gear contours are extracted, except for the inner edge right angles, with an exceptionally low error rate.

By extracting the gear boundaries and in conjunction with the data presented in Table 3, it is evident that the MARM performs optimally in terms of overall quality. The *α*-shape results are very close to those of the MARM, but with the possibility of sacrificing correct points to achieve a zero error rate. The normal method is the least effective, as most edges perpendicular to the normal axis are not extracted. In terms of computational time, the *α* method is the most efficient, requiring about 7 s, followed by our approach at about 80 s. The normal method, due to the large radius estimated for the normal phase, results in a total time of over 770 s.

Due to the significant difference in both quality and efficiency between the normal method and the other two methods, the normal method will not be included in the subsequent examples’ comparative analysis.

**Figure 21:**
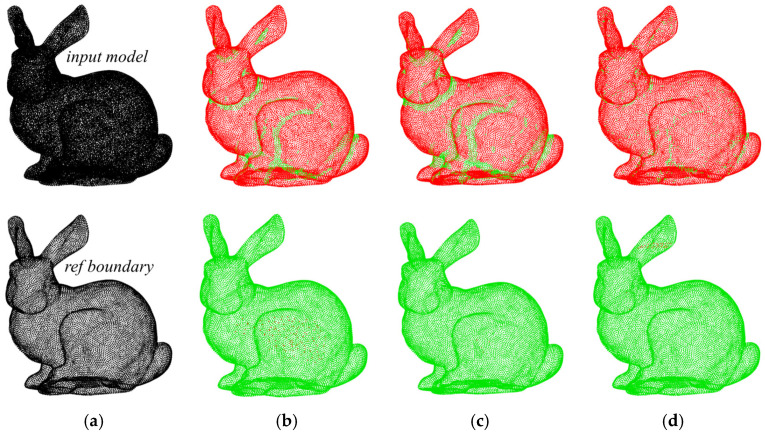
Boundary extraction results for the Stanford bunny: (**a**) the 3D input point cloud, with the upper image serving as the computational input and the lower image serving as the reference boundary for evaluating the results; (**b**) the *α*-shape result at *α* = 3*ρ*; (**c**) the *α*-shape result at *α* = 3.7*ρ*; (**d**) the result of the MARM at *r*_o_ = 2*ρ*.

We used both the *α*-shape and the MARM to extract the point cloud boundaries of the Stanford bunny for Figure 21a. As shown in Figure 21b, when *α* is set to 3*ρ*, the *α*-shape method fails to extract points at certain regions of high curvature (green areas in the upper panel); while inside these regions, notable non-boundary points are evident due to the sparse point cloud (red areas in the lower panel). To mitigate the presence of non-boundary points, we incrementally increased the value of *α*. As shown in Figure 21c, at *α* = 3.7*ρ*, only four erroneously extracted points remain internally but are accompanied by a significant reduction in the number of correctly identified points compared to Figure 21b. As shown in Figure 21d, the MARM, with the default setting *r*_o_ = 2*ρ*, produced the highest number of boundary points, with erroneous extraction occurring exclusively at the intricately curved regions of the ears, and only a few boundary points showing inaccuracies; nevertheless, the overall quality of extraction surpasses that of the *α*-shape method.

**Figure 22:**
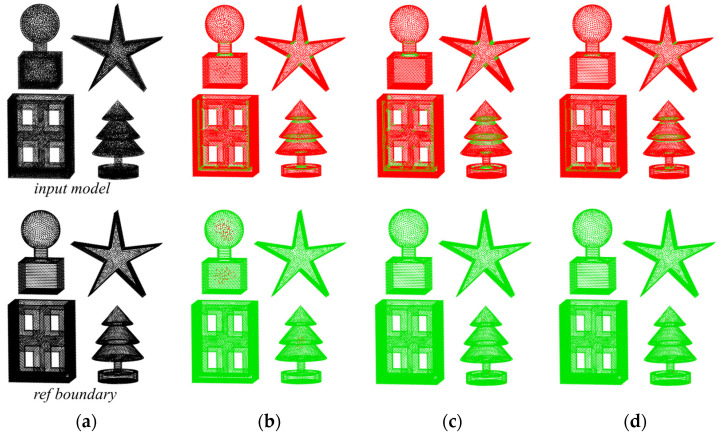
Boundary extraction results for the multi-body model: (**a**) the 3D input point cloud, with the upper image serving as the computational input and the lower image serving as the reference boundary for evaluating the results; (**b**) the *α*-shape result at *α* = 2*ρ*; (**c**) the *α*-shape result at *α* = 2.5*ρ*; (**d**) the result obtained with the MARM at *r*_o_ = 2*ρ*.

To assess the quality of boundary extraction in multi-body models, we used the *α*-shape and the MARM to extract the point cloud boundaries for Figure 22a. Figure 22b,c show the *α*-shape results at *α* = 2*ρ* and *α* = 2.5*ρ*, respectively. The extraction results in Figure 22b reveal more detail than those in Figure 22c, with fewer green segments in the upper panel and a higher accuracy of boundary extraction. However, there are more red points in the “seal” and “tree” regions in the lower part of Figure 22b, indicating that internal sparsity disturbs the extraction results. Figure 22d shows the results of the MARM, maintaining the parameter *r*_o_ = 2*ρ*. In terms of boundary extraction points, the unextracted areas align closely with the *α*-shape. Referring to Table 4, the MARM identifies marginally more correct points than the *α*-shape, albeit with a few isolated errors in each figure. Overall, the quality of extraction is superior to that of the *α*-shape.

#### 4.2.3. Application

**Figure 23:**
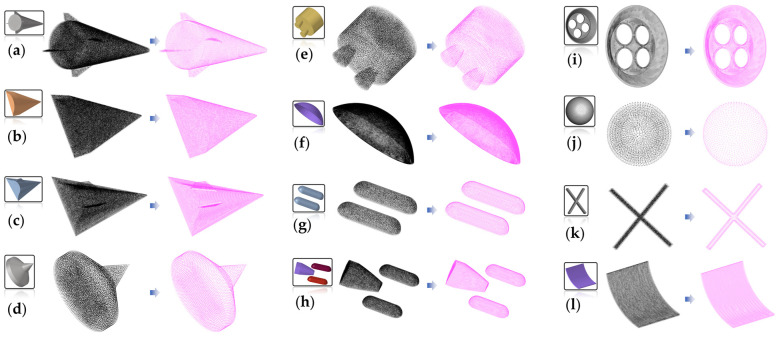
Extracting point cloud boundaries from some typical models in spacecraft re-entry calculations: (**a**–**d**) represent the entire model of the spacecraft, while (**e**–**l**) represent the fragmentation model during the re-entry process. The black points represent the input point clouds, and the magenta points represent the boundary results.

We attempted to extract boundaries on some spacecraft models to demonstrate that more structures can adapt to the finite element mesh boundary condition loading under the MARM, as shown in Figure 23.

Figure 23 shows the extraction of point cloud boundaries. The point cloud boundaries of the entire spacecraft model and the fragment model have been fully extracted. Among them, (g) and (h) belong to the split structure, which verifies that our boundary extraction method is fully capable of dealing with non-connected models; (i) belongs to a thin-walled structure, and the proportion of boundary point clouds is large, indicating that our method is not affected by the distribution proportion of boundary points.

We have recorded the corresponding *r*_o_ values and accuracy for the boundary extraction in Table 5. In Table 5, where the boundary accuracy is not lower than 96%, and the maximum extraction error does not exceed 0.3%, further verifying that the MARM effectively extracts the boundaries of irregular spacecraft and components. For the point cloud boundary extraction results in Table 5, combined with the original structural mesh data, the correspondence between points and meshes can be used to obtain the surface boundary mesh.

## 5. Conclusions

We presented a circle’s pure rolling method for the boundary extraction of 2D point clouds. On the basis of the circle rolling method, plane moving adsorption is carried out, forming the MARM that can handle the 3D point cloud; it can be applied to the acquisition of boundary meshes in finite element models of spacecraft structures. Our method for hole models and overlapping points has been improved such that boundary extraction does not lead to incomplete areas or infinite loops. Our method is compared with the *α*-shape algorithm:

(1) For the CRM (2D point cloud boundary extraction), we obtain the same high-quality boundary points and we do so faster, especially when dealing with ultra-large-scale point clouds, and our method is more resistant to interference from internal sparse points.
(2) For the MARM (3D point cloud boundary extraction), we are better at surface concavities and are not affected by internal sparse points.

Future work: Our method cannot handle the inner boundary problem at present. However, we need only design an algorithm that can find the first point of the inner boundary such that the scrolling circle can scroll on the inner boundary, or find a way to cut the point cloud of the inner boundary to create multiple regions containing only the outer boundary, and then extract, merge, and de-duplicate the boundary. The current value of *r*_o_ we use is a global density; if we switch to a local value based on the rolling position, with the rolling circle adaptively adjusting its size, the accuracy of boundary extraction will be further improved.

## Figures and Tables

**Table 1:** Comparison of boundary extraction accuracy and error rates of four types of point clouds under the CRM and α-shape. In which “*α* = x*ρ*” indicates the α-shape algorithm and “*r*_o_ = x*ρ*” indicates our method, with similar notation to follow.

Rate Type	Irregular		Smiling Face		Ratchet		Non-Connected	
	Factor	Value	Factor	Value	Factor	Value	Factor	Value
$k_{bdry}$	*α* = 6*ρ*	100%	*α* = 4*ρ*	98.0%	*α* = 2.4*ρ*	95.5%	*α* = 5*ρ*	98.1%
$k_{error}$		41.8%		0		41.7%		0
$k_{bdry}$	*α* = 10*ρ*	96.3%	*r*_o_ = 2*ρ*	52.3%	*α* = 4*ρ*	83.1%	*r*_o_ = 1.3*ρ*	98.1%
$k_{error}$		0		0		29.4%		0
$k_{bdry}$	*α* = 15*ρ*	92.7%	-	-	*α* = 4*ρ*	62.9%	-	-
$k_{error}$		0		-		0		-
$k_{bdry}$	*r*_o_ = 1.5*ρ*	96.3%	-	-	*r*_o_ = 0.5*ρ*	100%	-	-
$k_{error}$		0		-		0.4%		-
$k_{bdry}$	*r*_o_ = 2*ρ*	92.7%	-	-	*r*_o_ = 0.81*ρ*	96.6%	-	-
$k_{error}$		0		-		0		-

**Table 2:** Number of input points and time required for the two methods.

Input (pcs)	Results (pcs)	Time (s)			
		*α* = 20*ρ*	*α* = 200*ρ*	*r*_o_ = *ρ*	*r*_o_ = 2*ρ*
6561	320	0.371	0.382	0.076	0.148
40401	800	2.486	2.385	0.273	0.459
160,801	1600	9.838	9.732	0.837	1.194
641,601	3200	39.871	40.091	2.85	3.591
4,004,001	8000	260.56	253.225	16.617	18.383

**Table 3:** Results and time for Figure 20.

	Input Model	Ref Boundary	(b)	(c)	(d)	(e)	(f)
Results (pcs)	103,086	44,697	42,121	41,965	17,571	16,609	42,731
Time (s)	-	-	7.783	7.648	771.628	779.352	83.018

**Table 4:** Results and time for Figure 22.

	Input Model	Ref Boundary	(b)	(c)	(d)
Results (pcs)	56,382	28,208	26,152	25,281	26,842
Time (s)	-	-	6.176	5.947	61.91

**Table 5:** The corresponding *r*_o_ values and accuracy in boundary extraction for Figure 23.

Model	Input Model	*r*_o_	*k_cor_*	*k_error_*	Model	Input Model	*r*_o_	*k_cor_*	*k_error_*
(a)	195,897	1.5*ρ*	98.1%	0.2%	(g)	26,101	2*ρ*	99.6%	0
(b)	78,599	1.5*ρ*	99.4%	0	(h)	73,409	2*ρ*	99.2%	0
(c)	142,896	1.5*ρ*	97.3%	0	(i)	147,293	2*ρ*	96.8%	0.1%
(d)	71,483	1.5*ρ*	99.7%	0	(j)	17,794	2*ρ*	97.6%	0
(e)	108,729	0.9*ρ*	99.7%	0.3%	(k)	11,657	1.5*ρ*	99.5%	0
(f)	108,505	1.5*ρ*	98.7%	0	(l)	82,638	2*ρ*	1	0

## Data Availability

Data will be made available upon reasonable request.
